# Functional identification of SLC43A3 as an equilibrative nucleobase transporter involved in purine salvage in mammals

**DOI:** 10.1038/srep15057

**Published:** 2015-10-12

**Authors:** Junji Furukawa, Katsuhisa Inoue, Junya Maeda, Tomoya Yasujima, Kinya Ohta, Yoshikatsu Kanai, Tappei Takada, Hirotaka Matsuo, Hiroaki Yuasa

**Affiliations:** 1 Department of Biopharmaceutics, Graduate School of Pharmaceutical Sciences, Nagoya City University, Nagoya, Japan; 2 Department of Biopharmaceutics, School of Pharmacy, Tokyo University of Pharmacy and Life Sciences, Tokyo, Japan; 3 Division of Bio-system Pharmacology, Department of Pharmacology, Graduate School of Medicine, Osaka University, Osaka, Japan; 4 Department of Pharmacy, The University of Tokyo Hospital, Tokyo, Japan; 5 Department of Integrative Physiology and Bio-Nano Medicine, National Defense Medical College, Saitama, Japan

## Abstract

The purine salvage pathway plays a major role in the nucleotide production, relying on the supply of nucleobases and nucleosides from extracellular sources. Although specific transporters have been suggested to be involved in facilitating their transport across the plasma membrane in mammals, those which are specifically responsible for utilization of extracellular nucleobases remain unknown. Here we present the molecular and functional characterization of SLC43A3, an orphan transporter belonging to an amino acid transporter family, as a purine-selective nucleobase transporter. SLC43A3 was highly expressed in the liver, where it was localized to the sinusoidal membrane of hepatocytes, and the lung. In addition, SLC43A3 expressed in MDCKII cells mediated the uptake of purine nucleobases such as adenine, guanine, and hypoxanthine without requiring typical driving ions such as Na^**+**^ and H^**+**^, but it did not mediate the uptake of nucleosides. When SLC43A3 was expressed in APRT/HPRT1-deficient A9 cells, adenine uptake was found to be low. However, it was markedly enhanced by the introduction of SLC43A3 with APRT. In HeLa cells, knock-down of SLC43A3 markedly decreased adenine uptake. These data suggest that SLC43A3 is a facilitative and purine-selective nucleobase transporter that mediates the cellular uptake of extracellular purine nucleobases in cooperation with salvage enzymes.

Nucleotides play vital roles in all living organisms both by forming the nucleic acids DNA and RNA, which store and implement genetic information, and as individual monomers involved in biological signaling and energy cycling. The cellular pool of such nucleotides is strictly regulated by catabolism and biosynthesis; the latter employs both *de novo* and salvage pathways. Although the *de novo* pathway assembles purine and pyrimidine nucleotides from several fundamental molecules such as amino acids and glucose through multistep reactions, including those that produce nucleobases and nucleosides before their conversion to nucleotides, the salvage pathway performs the same task by simply reutilizing the nucleobases and nucleosides that are produced by the degradation of nucleotides within cells^1^. Nucleobases and nucleosides can also be salvaged extracellularly from dietary sources and from some tissues that produce excess nucleobases and nucleosides via the *de novo* pathway, thus supplementing the rather limited supply of degraded nucleotide products. In particular, in many types of cells exhibiting poor *de novo* synthesis activity, the salvage pathway has been suggested to play a major role in nucleotide production.

Extracellularly supplied nucleobases and nucleosides must be transported across the plasma membrane to be utilized by the salvage pathway. Because this process is generally difficult for this class of hydrophilic compounds to undergo by simple diffusion, the involvement of specific transporters has been suggested. For nucleosides, concentrative nucleoside transporters (CNTs/SLC28As) and equilibrative nucleoside transporters (ENTs/SLC29As) have been identified as involved in this process and their transport functions have been well characterized^2^,^3^,^4^,^5^. The CNT family includes CNT1/SLC28A1 and CNT2/SLC28A2, which operate unidirectionally for influx of nucleosides by a sodium-dependent secondary active mechanism at the apical membrane of the epithelial cells in several organs, typically the small intestine and kidney; the ENT family includes ENT1/SLC29A1 and ENT2/SLC29A2, which bidirectionally facilitate influx and efflux depending on the substrate concentration gradient at the basolateral membrane in epithelial cells of the same organs. The CNT family has one more member, CNT3/SLC28A3, which is expressed mainly in pancreas, trachea, bone marrow, and mammary gland. The ENT family has two more members, ENT3/SLC29A3 and ENT4/SLC29A4, both of which are expressed in a wide variety of tissues and operate in a pH-dependent manner. The former is a nucleoside transporter that operates at the lysosomal membrane, and the latter is redefined as plasma membrane monoamine transporter (PMAT), which operates multispecifically for the transport of monoamines and some other types of cationic compounds, as well as adenosine.

However, the molecular mechanisms underlying nucleobase transport are unresolved in mammals, although nucleobase transport systems have been suggested to occur in various cells and tissues. These hypothetical transport systems can be classified into secondary active transporters, which are coupled with sodium, and facilitative transporters, just as nucleoside transporters are classified. ENT1 and ENT2 can mediate the transport of purine nucleobases, such as adenine, hypoxanthine, and guanine, in a facilitative manner^6^,^7^; however, purine transport by ENTs, which can be inhibited by nucleosides, their principal substrates, cannot account for the purine nucleobase-selective transport system. Such a system has been observed in red blood cells, where it is involved in the facilitative uptake of adenine, which is inhibited competitively by hypoxanthine but not by nucleosides. Similar purine nucleobase-selective transport systems have been suggested to be present in several types of cells, such as the cell lines L929^8^, CHO^8^, and COR-L23^9^, as well as in rabbit cornea epithelial cells^10^ and human cardiac microvascular endothelial cells^11^. These transport systems, such as those in red blood cells, are sodium independent, highly nucleobase selective in substrate recognition, and insensitive to specific inhibitors of ENTs. Furthermore, the facilitative uptake of hypoxanthine and adenine has been observed even in cell lines that lack nucleoside transport systems^12^. These lines of evidence strongly suggest the presence of one or more facilitative nucleobase transporters in addition to ENTs. Although we have recently identified sodium-dependent nucleobase transporter 1 (SNBT1/Slc23a3) in rat as the first nucleobase transporter in mammals, this transporter differs from such facilitative nucleobase transporters in its sodium-dependent function and ability to transport uracil and guanine and their analogs^13^. Moreover, SNBT1 is pseudogenized in humans and some other higher primates. Thus, at least one facilitative nucleobase-specific transporter remains unidentified in mammals.

We here report that SLC43A3, an orphan transporter that is abundantly expressed in the liver and lung, can mediate the transport of purine nucleobases specifically. Based on the facilitative nature in its function, we have named it equilibrative nucleobase transporter 1 (ENBT1). A detailed functional analysis has shown that salvage enzymes cooperate in the cellular uptake of purine nucleobases mediated by ENBT1.

## Results

### Identification of SLC43A3 as a nucleobase transporter

**Figure 1:**
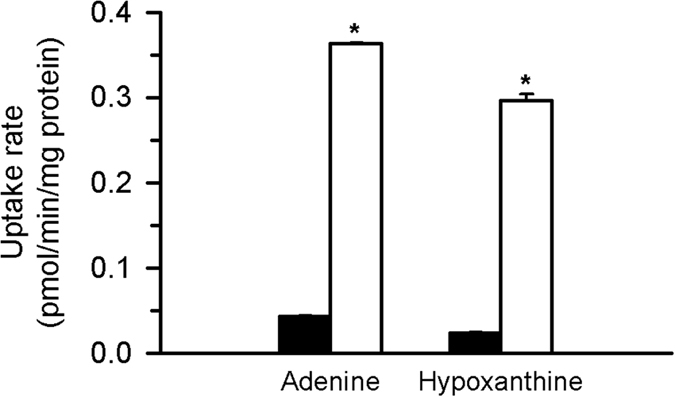
ENBT1 mediates the uptakes of adenine and hypoxanthine. The uptakes of [^3^H]adenine and [^3^H]hypoxanthine (5 nM for each) were evaluated at 37 °C and pH 7.4 for 1 min in HEK293 cells transiently expressing ENBT1 (open bars) and mock cells (filled bars) for control. *p < 0.05, *n* = 4.

To identify a candidate gene for purine nucleobase transport function, we conducted a comprehensive screen of human cDNA encoding putative transporters based on the uptake activity of [^3^H]adenine in HEK293 cells transiently expressing each cDNA. Among these cDNA, we observed that the clone encoding SLC43A3 induced an increase in the cellular uptake of [^3^H]adenine (Fig. 1). This purine nucleobase transport activity was also observed for [^3^H]hypoxanthine.

SLC43A3 is an orphan transporter belonging to an amino acid transporter family comprising three members, along with LAT3/SLC43A1^14^ and LAT4/SLC43A2^15^, both of which mediate the uptake of neutral amino acids such as leucine. Human SLC43A3 is 491 amino acid residues in length and predicted to have 12 transmembrane domains. The amino acid sequence of SLC43A3 is ~27% identical with those of LAT3 and LAT4 but not significantly similar to those of any other transporters, including nucleoside transporters. The orthologs of SLC43A3 are conserved among several mammalian species.

### Functional characterization of SLC43A3

**Figure 2:**
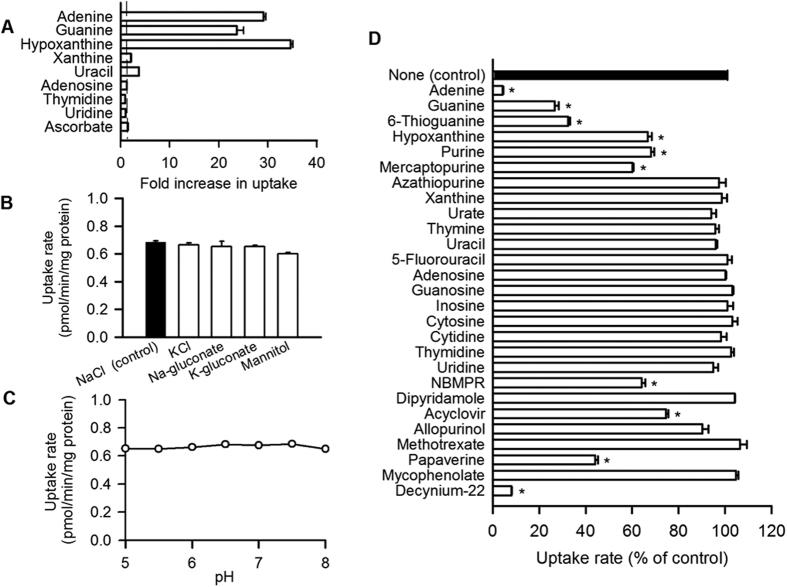
Functional characterization of ENBT1 stably expressed in MDCKII cells. (**A**) Substrate specificity of ENBT1. ENBT1-mediated uptake was evaluated by dividing the uptake in the cells expressing ENBT1 by that in mock cells. The uptakes of ^3^H-labeled adenine, guanine, hypoxanthine, xanthine, uracil, adenosine, thymidine, and uridine (10 nM for guanine and 5 nM for the others), and ^14^C-labeled ascorbate (50 μM) were evaluated at 37 °C for 1 min. The broken line is for unity. (**B**) Effect of extracellular ions on the ENBT1-specific uptake of [^3^H] adenine. NaCl in the control medium was replaced as indicated. The uptake of [^3^H]adenine (5 nM) was evaluated at 22 °C for 40 s in the cells expressing ENBT1 and mock cells, and the ENBT1-specific uptake was calculated by subtracting the uptake in the latter from that in the former. (**C**) Effect of extracellular pH on the ENBT1-specific uptake of [^3^H] adenine. The uptake of [^3^H]adenine (5 nM) was evaluated at 22 °C for 40 s. (**D**) Effect of various compounds on the ENBT1-specific uptake of [^3^H]adenine. The uptake of [^3^H]adenine (5 nM) was evaluated at 22 °C for 40 s in the presence of an inhibitor (10 μM for decynium-22 and 200 μM for the others) or in its absence. *p < 0.05, *n* = 4.

To characterize the transport function of SLC43A3 in detail, we generated MDCKII cells stably expressing human SLC43A3 (henceforth, SLC43A3 cells) and examined the uptake of various nucleobases and nucleosides in these cells. The uptake of purine nucleobases such as [^3^H]guanine and [^3^H]hypoxanthine as well as [^3^H]adenine were markedly greater in SLC43A3 cells than in mock cells, consistent with the purine nucleobase uptake of the HEK293 cells that transiently expressed SLC43A3, further suggesting that this transporter is a purine nucleobase transporter (Fig. 2A). Although [^3^H]xanthine is also a purine nucleobase, SLC43A3 cells demonstrated only minimal uptake of this nucleobase. Cellular uptake of [^3^H]uracil, a pyrimidine nucleobase, was also observed to be minimal in SLC43A3 cells. Nucleosides, such as [^3^H]adenosine, [^3^H]thymidine, and [^3^H]uridine, did not exhibit increased uptake by SLC43A3 cells. Ascorbate was also included as a test compound because mammalian ascorbate transporters are orthologs of bacterial nucleobase transporters^16^, but this compound did not exhibit increased uptake by SLC43A3 cells. Among those compounds that appeared to be transported by SLC43A3, we selected adenine to be used as a probe substrate for further assessment of the transport function of SLC43A3 as it is one of the best characterized nucleobases exhibiting carrier-mediated transport in biological systems.

The specific uptake of [^3^H]adenine by SLC43A3 cells was not altered by replacement of NaCl in the medium with any of the sodium or potassium salt of chloride or gluconate or mannitol, suggesting that this transporter does not require extracellular Na^+^ or Cl^−^ for operation (Fig. 2B). In addition, uptake was not altered by changes in pH throughout a range of 5.0–8.0, suggesting that H^+^ is not required for its operation either (Fig. 2C). These results support the role of SLC43A3 as a facilitative nucleobase transporter permitting substrate nucleobases to rapidly equilibrate across the plasma membrane; accordingly, we named it ENBT1. This transporter also appears to transport purine nucleobases preferentially to uracil as a pyrimidine nucleobase, as suggested in Fig. 2A.

### Effect of various compounds on ENBT1-mediated adenine transport

To further explore the substrate specificity of ENBT1, we examined the effect of various compounds (10 μM for decynium-22 and 200 μM for the others) on the ENBT1-mediated uptake of [^3^H]adenine in SLC43A3 cells (Fig. 2D). Purine nucleobases such as adenine, guanine, and hypoxanthine, which were found to be substrates of SLC43A3, and hence, potential competitive inhibitors, inhibited [^3^H]adenine uptake of SLC43A3 cells. Moreover, purine and its several analogs, such as 6-thioguanine and mercaptopurine, showed inhibitory activities, suggesting that they are also substrates. In contrast, xanthine, a purine nucleobase, and uracil, a pyrimidine nucleobase, did not exhibit any substantial inhibitory activity. Because these two molecules were transported by SLC43A3 minimally (Fig. 2A), they are likely to be recognized as very poor substrates with affinities extremely low to act as competitive inhibitors. The other pyrimidine nucleobases (thymine and cytosine) and 5-fluorouracil, an analog of uracil, did not exhibit any inhibitory activity either. Adenosine, a purine nucleoside, and thymidine and uridine, which are pyrimidine nucleosides, did not exhibit any inhibitory activity, consistent with the finding that they are not transported by SLC43A3 (Fig. 2A). Several other purine and pyrimidine nucleosides did not exhibit any inhibitory activity either. Thus, ENBT1 did not recognize any purine or pyrimidine nucleosides as substrates (or competitive inhibitors). Nitrobenzylthioinosine (NBMPR), a potent inhibitor of ENT1, exhibited inhibitory activity, although its inhibition was not extensive. Dipyridamole, a potent inhibitor of both ENT1 and ENT2, did not exhibit inhibitory activity. Decynium-22, which has recently been reported as a specific inhibitor of a purine nucleobase transport system in PK15NTD cells^17^, was found to be a potent ENBT1 inhibitor that can inhibit [^3^H]adenine uptake almost completely at 10 μM. We also examined the effect of various amino acids (5 mM), as ENBT1 is a member of the amino acid transporter family^14^,^15^; however, none of them affected the adenine uptake activity of SLC43A3 cells (see Supplementary Table S1 online), supporting a previous study indicating that SLC43A3 expression did not induce any amino acid transport activity in *Xenopus* oocytes^18^.

### Kinetic analysis

**Figure 3:**
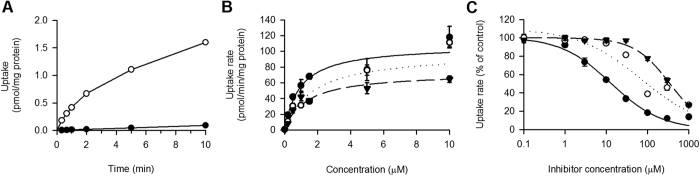
Kinetic analyses of the uptakes of purine nucleobase mediated by ENBT1 stably expressed in MDCKII cells. (**A**) Time courses of the uptake of [^3^H] adenine (5 nM) was evaluated at 22 °C in the cells expressing ENBT1 (open circle) and mock cells (closed circle). (**B**) Concentration-dependent uptakes of adenine (•), guanine (○) and hypoxanthine (▼) were evaluated at 22 °C for 40 s in the cells expressing ENBT1 and mock cells. The ENBT1-specific uptake was calculated by subtracting the uptake in the latter from that in the former, and was used for kinetic analysis. The values of K_m_ (μM) are 0.94 ± 0.14, 1.70 ± 0.54, and 1.32 ± 0.26 for adenine, guanine, and hypoxanthine, respectively, as the computer-fitted parameters with S.E. (*n* = 7–8), and those of V_max_ (pmol/min/mg protein) are 107.8 ± 9.6, 98.2 ± 20.7, and 73.1 ± 9.2, respectively. Solid line, dotted line, and dashed line are computer-fitted profiles for adenine, guanine, and hypoxanthine, respectively. (**C**) Concentration-dependent inhibition of the ENBT1-mediated uptake of [^3^H]hypoxanthine by adenine (•), and that of [^3^H]adenine by guanine (○) and hypoxanthine (▼). The uptakes of [^3^H]hypoxanthine and [^3^H]adenine (5 nM for each) were evaluated at 22 °C for 40 s in the presence of a nucleobase as an inhibitor at specified concentrations or in its absence. The values of IC_50_ (μM) are 13.0 ± 3.7, 70.0 ± 46.3, and 350.0 ± 31.2 for adenine, guanine, and hypoxanthine, respectively, as the computer-fitted parameters with S.E. (*n* = 8). Solid line, dotted line, and dashed line are computer-fitted profiles for adenine guanine, and hypoxanthine, respectively. All the data are presented as the means ± S.E. (*n* = 4).

The time courses of [^3^H]adenine uptake were examined in both SLC43A3 cells and mock cells (Fig. 3A). The [^3^H]adenine uptake increased rapidly, exhibiting a linear increase for up to 1 min in SLC43A3 cells, whereas it was far slower in mock cells. Accordingly, we decided to evaluate the initial rate of nucleobase uptake at 40 s. Kinetic analysis indicated that the specific uptakes of [^3^H]adenine, [^3^H]guanine, and [^3^H]hypoxanthine were saturable with the K_m_ values of 0.94 μM, 1.70 μM, and 1.32 μM, respectively (Fig. 3B). However, as shown in Fig. 3C, the adenine IC_50_ value for [^3^H]hypoxanthine uptake (13 μM), and the guanine and hypoxanthine IC_50_ values for [^3^H]adenine uptake (70 μM and 350 μM, respectively) were greater than the K_m_ values for the respective nucleobases.

When a substrate acts as a competitive inhibitor, its IC_50_ should be theoretically comparable to its inhibition constant (K_i_) and its transport constant (K_m_) as long as IC_50_ is determined for the inhibition of the transport of a test substrate at a concentration much lower than its K_m_, as in the present study. Based on this, the discrepancies between K_m_ and IC_50_ are unexpected findings.

However, this inconsistency can be explained by considering that each of the [^3^H]-labeled nucleobases is converted to its nucleotide by its specific salvage enzyme (adenine phosphoribosyltransferase (APRT) for adenine and hypoxanthine phosphoribosyltransferase 1 (HPRT1) for guanine and hypoxanthine) in SLC43A3 cells and thus accumulated in that form. This may maintain low concentrations of nucleobase forms within the cells, thus sustaining its inwardly directed concentration gradient across the plasma membrane, which is the driving force for facilitative transport mediated by ENBT1. Therefore, the kinetic parameters of the apparent uptake of [^3^H]-labeled nucleobases may be aggregates of the cooperative process that involves ENBT1 and the specific salvage enzymes. Because the K_m_ of [^3^H]adenine uptake (0.94 μM) was comparable to those of APRT for adenine in the literature (0.6–0.9 μM), apparent [^3^H]adenine uptake may be rate-limited by APRT-mediated metabolism. Similarly, the K_m_ of [^3^H]hypoxanthine uptake (1.32 μM) was comparable to that of HPRT1 for hypoxanthine (3 μM), suggesting that apparent [^3^H]hypoxanthine uptake could be rate-limited by HPRT1-mediated metabolism. On the other hand, within experiments evaluating the adenine IC_50_ value for [^3^H]hypoxanthine uptake, adenine acts on ENBT1 but not HPRT1. Therefore, the empirical IC_50_ value (13 μM) may represent the affinity of ENBT1 for adenine. This IC_50_ value was equal to the K_m_ value for adenine uptake in human erythrocytes (also 13 μM^19^), which is likely to represent the transporter affinity. In addition, the IC_50_ value is much lower than the K_m_ values of ENT1 (3.2 mM) and ENT2 (1.8 mM) for adenine in the literature^6^,^7^ and closer to adenine concentrations in plasma (~1 μM), suggesting that ENBT1 operates more effectively at physiologically relevant adenine concentrations than ENTs. Similarly, the guanine and hypoxanthine IC_50_ values (70 and 350 μM, respectively) for [^3^H]adenine uptake are likely to represent the affinity of guanine and hypoxanthine for ENBT1 because they act on ENBT1 but not APRT.

### Cooperative operation of ENBT1 with salvage enzymes

**Figure 4:**
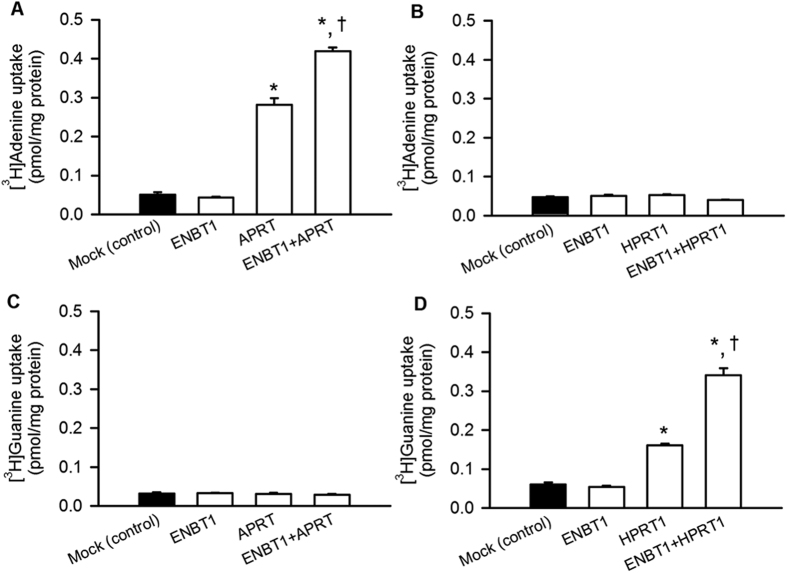
Cooperative operation of ENBT1 with salvage enzymes for purine nucleobases. The uptake of [^3^H]adenine (5 nM) was evaluated at 37 °C for 1 min in APRT/HPRT1-deficient A9 cells transfected with a plasmid for ENBT1 and one for APRT (**A**) or HPRT1 (**B**) with 1: 1 ratio (1 μg of total plasmid). The uptake of [^3^H]guanine (5 nM) was evaluated similarly in A9 cells transfected with a plasmid for ENBT1 and one for APRT (**C**) or HPRT1 (**D**). *Significantly different from control at *p* < 0.05. ^†^Significantly different from APRT or HPRT1 alone at *p* < 0.05. All the data are presented as the means ± S.E. (*n* = 3 or 4).

To explore the effect of such salvage enzymes on nucleobase uptake mediated by ENBT1, we conducted a series of uptake experiments with mouse fibroblast-derived A9 cells, which are deficient in APRT and HPRT1. The uptake of [^3^H]adenine in A9 cells was low and not enhanced by the introduction of human ENBT1. However, the introduction of both human APRT and ENBT1 resulted in a significant increase in [^3^H]adenine uptake, suggesting their cooperation in adenine uptake (Fig. 4A). Although [^3^H]adenine uptake was also increased by the introduction of APRT alone, the increase could be due to the involvement a native nucleobase transporter present in A9 cells. The introduction of human HPRT1, instead of APRT, into A9 cells did not induce any increase in [^3^H]adenine uptake, regardless of the presence or absence of cointroduced ENBT1 (Fig. 4B), suggesting the necessity of APRT for adenine uptake in cooperation with ENBT1. It should be noted additionally that [^3^H]adenine uptake increased in proportion to time at least up to 2 min in A9 cells expressing APRT alone and those expressing APRT with ENBT1, indicating that the uptake period of 1 min was in that range and appropriate for the evaluation of initial uptake process (see Supplementary Fig. S2 online). Although [^3^H]adenine uptake in A9 cells expressing ENBT1 alone was too small to confirm such an initial uptake phase, [^3^H]adenine uptake was greater in A9 cells expressing APRT with ENBT1 than in those expressing ENBT1 alone at the earliest time of 20 s and linearly increased with time for a more prolonged period in the former than in the latter. Therefore, it is most likely that APRT worked for accelerating and enhancing [^3^H]adenine uptake by metabolic channelling in cooperation with ENBT1. Similarly to the results for [^3^H]adenine uptake, [^3^H]guanine uptake was enhanced by the introduction of HPRT1 alone and furthermore by the additional introduction of ENBT1, whereas it was not enhanced in any A9 cells with APRT or ENBT1 alone (Fig. 4C,D). Thus, HPRT1 appears to be necessary for guanine uptake in cooperation with ENBT1.

**Figure 5:**
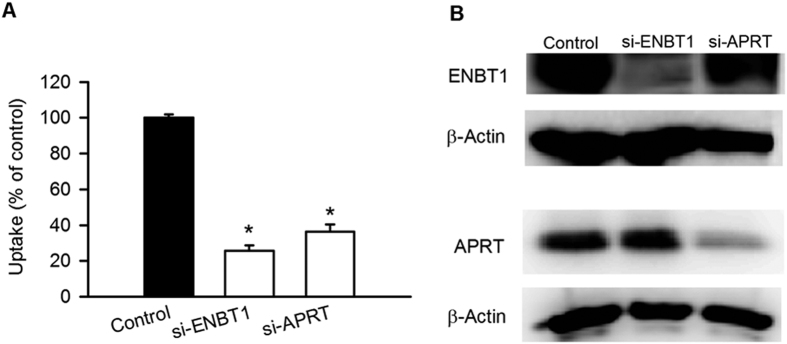
Effect of silencing of ENBT1 and APRT on [^3^H]adenine uptake in HeLa cells. (**A**) The uptake of [^3^H]adenine (5 nM) was evaluated at 37 °C for 1 min in HeLa cells transfected with siRNA for ENBT1 or APRT. *p < 0.05, *n* = 4. (**B**) The endogenous protein levels for ENBT1, APRT and β-actin in those cells were analyzed by western blotting.

Similarly, silencing ENBT1 or APRT by RNA interference in HeLa cells, which exhibit native adenine uptake, substantially decreased [^3^H]adenine uptake (Fig. 5A), in accordance with reduced expression levels of each protein (Fig. 5B).

Collectively, these results suggest that ENBT1 is involved in the utilization of extracellular purine nucleobases through functional interactions with salvage enzymes.

### Tissue distribution and cellular localization of ENBT1

**Figure 6:**
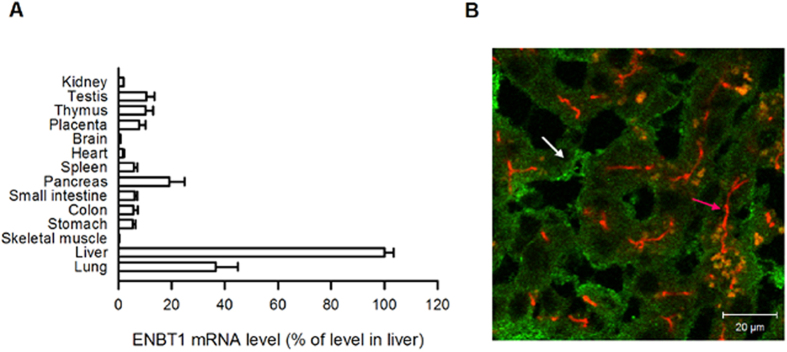
Tissue distribution and cellular localization of ENBT1. (**A**) The mRNA level of ENBT1 was determined by real-time PCR and standardized by the mRNA level of GAPDH in each human tissue (*n* = 4). (**B**) The immunofluorescent image shows the subcellular localization of ENBT1 (green, white arrow) and P-glycoprotein (red, pink arrow) in the human liver.

To examine the expression pattern of ENBT1 in human tissues, we performed quantitative real-time RT-PCR. ENBT1 mRNA was expressed in all the tissues examined, although at the highest levels in the liver and lung, followed by the pancreas (Fig. 6A).

Immunofluorescence staining of human liver showed that ENBT1 was localized to the basolateral (sinusoidal) membrane of human hepatocytes and was clearly distinguished from P-glycoprotein, which localized to the apical (canalicular) membrane (Fig. 6B). Basolateral localization of ENBT1 was also observed by confocal laser scanning microscopy in polarized MDCKII cells stably expressing ENBT1 fused with green fluorescent protein (see Supplementary Fig. S1 online).

## Discussion

SLC43A3 was originally identified as embryonic epithelia gene 1 (EEG1), a gene upregulated in a cell culture model of kidney development^20^. Because its mRNA is also expressed in several embryonic tissues, such as lung and liver, and its peak expression occurs during embryogenesis, it has been implicated in the processes of cellular proliferation and tissue development. In the present study, we have demonstrated that ENBT1/SLC43A3 is a facilitative and purine-selective nucleobase transporter that mediates the cellular uptake of extracellular purine nucleobases, a key step for their efficient utilization in the salvage pathway. This is the first report that has identified a purine-specific nucleobase transporter in mammals.

The presence of at least three types of transport systems for the uptake of hypoxanthine in human has been postulated based on differences in sensitivity to inhibitors^21^. Two of these hypothetical transports are inhibited by nucleosides competitively and also by dipyridamole; in conjunction with recent ENT studies, ENT1 and ENT2 have been identified as responsible for one of the mechanisms inhibited by NBMPR and the other NBMPR-insensitive mechanism^6^,^7^. The third system is not inhibited by either NBMPR or dipyridamole, but it is competitively inhibited by adenine but not nucleosides. These characteristics are in agreement with those of ENBT1, suggesting that ENBT1 is the transporter responsible for this nucleobase uptake system. Although adenine transport by ENBT1 was inhibited by NBMPR by approximately 40% at a 200 μM concentration (Fig. 2D), this fact implies that ENBT1 is far less sensitive to this inhibitor than ENT1, which is inhibited with a K_i_ of 2 nM.

The hypoxanthine uptake of human cardiac microvascular endothelial cells has been previously shown to be insensitive to NBMPR and dipyridamole but extensively inhibited by adenine with an IC_50_ value of 19 μM^11^, which is comparable with that of adenine IC_50_ for ENBT1 in the present study. Furthermore, a recent transcriptome analysis has revealed endothelial-specific SLC43A3 expression in broad types of microvasculatures^22^. As such, ENBT1 is likely to be functionally expressed in microvascular endothelial cells, where it may play an important role in the formation and homeostasis of blood vessels by regulating the cellular uptake of purine nucleobases. Furthermore, significant increases in SLC43A3 mRNA expression levels have been reported in the monocytes from blood of healthy volunteers infused with lipopolysaccharide^23^ and in thyroid carcinomas from patients exposed to radioactive iodine^24^, suggesting pathophysiological roles of ENBT1 in inflammation and carcinoma.

Among organs, the liver is the major one having *de novo* purine nucleobase synthesis activity. Therefore, high ENBT1 expression in the liver likely produces a net efflux of synthesized purine nucleobases, which could then be supplied to the other organs via the systemic circulation. For example, the lung receives all the systemic venous blood gathered to the heart, and hence, may receive all the nucleobases from the liver before they are distributed to the other organs through the systemic arteries. Moreover, the lung is rich in salvage enzymes that can produce nucleotides from nucleobases in cooperation with ENBT1, whereas it contains relatively less amount of *de novo* enzymes^25^,^26^. Therefore, high ENBT1 expression in the lung potentially enables the uptake of nucleobases from the liver, and the cooperative operation of ENBT1 and salvage enzymes could be the major mechanism for the production of nucleotides in the lung, which includes pulmonary endothelial cells, alveolar epithelial cells, and residential lymphoid cells. This would be of particular importance in the repair and growth of lung tissue injured by oxidative stress, which may be induced by respiratory exposure to oxygen. Lung injury is often caused by an adverse event associated with the use of several drugs, such as methotrexate and leflunomide, which act on cells actively synthesizing nucleotides^27^, including hepatocytes, by inhibiting *de novo* nucleotide synthesis; a reduction of nucleobases supplied by the liver may be indirectly involved in this damage. Thus, it is likely that the lungs rely heavily on the supply of nucleobases from the liver to maintain their function and that ENBT1 plays a critical role in their trafficking.

On the other hand, Bodoy *et al.*^18^ recently demonstrated that mice harboring a nonsense mutation in *Slc43a3* are phenotypically normal, without any sign of embryonic lethality. Other transporters, including nucleoside transporters, may have compensated for the functional defect of ENBT1. Further studies are needed to elucidate the role of ENBT1 in the utilization of purine nucleobases and homeostasis of nucleotides *in vivo*.

In conclusion, we have successfully identified the function of SLC43A3 as a novel, facilitative, purine-selective nucleobase transporter and demonstrated its cooperative operation with salvage enzymes in the cellular utilization of purine nucleobases. The functional characteristics of ENBT1 were observed to be almost completely in agreement with those of the previously described carrier-mediated transport system involved in the uptake of adenine and hypoxanthine in many types of cells and tissues. Therefore, it is most likely that SLC43A3 is its molecular entity. This transporter is apparently responsible for the cellular uptake of extracellularly supplied purine nucleobases, facilitating their salvage and subsequent use in the production of nucleotides. The present study provides new insights into the complexity of intracellular nucleotide level homeostasis and could lead to opportunities to develop new classes of nucleobase analogs for the treatment of cancer and viral infection, and also optimal therapeutic strategies for such diseases.

## Methods

### Materials

[^3^H]adenine (25.0 Ci/mmol), [^3^H]hypoxanthine (27.0 Ci/mmol), [^3^H]thymidine (20.0 Ci/mmol), [^3^H]uridine (14.7 Ci/mmol), [^3^H]uracil (42.8 Ci/mmol), [^3^H]xanthine (12.8 Ci/mmol) and [^3^H]adenosine (39.2 Ci/mmol) were obtained from Moravek Biochemicals (Brea, CA), [^14^C]guanine (55 mCi/mmol) was from American Radiolabeled Chemicals (St. Louis, MO), and [^14^C]ascorbate (8.5 mCi/mmol) was from PerkinElmer Life Sciences (Boston, MA). Adenine, papaverine, and dipyridamole were obtained from Sigma-Aldrich (St. Louis, MO), and hypoxanthine, guanine and nitrobenzylthioinosine were from Wako Pure Chemicals (Osaka, Japan). All other reagents were of analytical grade and commercially obtained.

### Cell culture

HEK293, HeLa, A9 and MDCKII cells were maintained at 37 °C and 5% CO_2_ in Dulbecco’s modified Eagle’s medium supplemented with 10% fetal bovine serum, 100 units/ml penicillin, and 100 μg/ml streptomycin.

### Preparation of HEK293 cells transiently expressing ENBT1

HEK293 cells (1.5 × 10^5^ cells/well initially) were grown on 24-well plates coated with poly-L-lysine for 12 h, transfected with 1 μg/well of the plasmid carrying the cDNA of ENBT1 by using 1.5 μl/well of Lipofectamine 2000 (Invitrogen, Carlsbad, CA) as a transfection reagent, according to the manufacturer’s instructions, and cultured for 36 h for transient expression.

### Transient expression of ENBT1, APRT and HPRT1 in A9 cells

For the transient expression of ENBT1 with APRT or HPRT1, A9 cells (2.0 × 10^5^ cells/well initially) were seeded on 24-well plates, transfected with the plasmid carrying the cDNA of ENBT1 and the one carrying the cDNA of APRT or HPRT1 by using Lipofectamine LTX (Invitrogen) as a transfection reagent, according to the manufacturer’s instructions, and cultured for 48 h. The cells were transfected with 1 μg/well of total plasmids with a ratio of 1:1 for the one for ENBT1 and the one for APRT, or HPRT1. For the expression of one of them alone, a half of the total amount of plasmids was replaced with the empty pCI-neo vector.

### Preparation of MDCKII cells stably expressing ENBT1

MDCKII cells were transfected with the plasmid carrying the cDNA of ENBT1 by using Lipofectamine 2000 and cultured in DMEM supplemented with 10% FBS and 800 μg/ml Geneticin for 2 to 3 weeks. Antibiotic-resistant clones were selected and tested for the transport of [^3^H]adenine as a probe substrate.

### Silencing of ENBT1 and APRT in HeLa cells

HeLa cells (2.5 × 10^4^ cells/well initially) were grown on 24-well plates coated with poly-L-lysine for 6 h, transfected with 4 pmol/well of the siRNA (StealthSelect RNAi^TM^, Invitrogen) specific to the mRNA of ENBT1 or APRT by using 0.5 μl/well of Lipofectamine RNAi MAX (Invitrogen), according to the manufacturer’s instructions, and cultured for 72 h for silencing of ENBT1 or APRT. For control, Stealth RNAi^TM^ Negative Control Low GC Duplex (Invitrogen) was used. The sequences of siRNA were as follows: ENBT1 sense, 5′- AAU GUC AGA AGA AUG GCA AGC AUG A -3′; ENBT1 antisense, 5′- UCA UGC UUG CCA UUC UUC UGA CAU U -3′; APRT sense, 5′-AGA UGU CCC UGA AUA CCA CGC CUG G-3′; APRT antisense, 5′- CCA GGC GUG GUA UUC AGG GAC AUC U -3′.

### Transport Study

MDCKII cells stably expressing ENBT1 (1.5 × 10^5^ cells/well initially) were grown on 24-well plates for 72 h. The cells in each well were preincubated in 0.25 ml of substrate-free uptake buffer (140 mM NaCl, 5 mM KCl, 0.4 mM KH_2_PO_4_, 0.8 mM MgSO_4_, 1.0 mM CaCl_2_, 25 mM glucose, and 10 mM HEPES, pH 7.4) for 5 min, unless otherwise indicated. Uptake assays were started by replacing the substrate-free uptake buffer for preincubation with one containing the radioisotope-labeled substrate (0.25 ml). All of the procedures were conducted at 22 °C or 37 °C. Assays were stopped by addition of ice-cold substrate-free uptake buffer (2 ml), and the cells were washed two times with 2 ml of the same buffer. The cells were solubilized in 0.5 ml of 0.2 M NaOH solution containing 0.5% SDS at room temperature for 1 h, and the associated radioactivity was measured by liquid scintillation counting. Cellular protein content was determined by the method of Lowry *et al.*^28^ using bovine serum albumin as the standard. Uptake assays were also conducted in mock cells, which were transfected with empty pCI-neo vector, to estimate nonspecific uptake.

Uptake assays were similarly conducted in HEK293 cells transiently expressing ENBT1, A9 cells transiently expressing ENBT1 and/or APRT, or HPRT1, and HeLa cells treated for silencing ENBT1 or APRT.

### Quantification of ENBT1 mRNA by real-time PCR

Real-time quantitative PCR was carried out using a THUNDERBIRD SYBR qPCR Mix (Toyobo) on a 7300 Fast Real-Time PCR System (Applied Biosystems, Forest City, CA). The expression levels were normalized with that of GAPDH. The sequences of primers were as follows: ENBT1 forward primer, 5′- ATT TTC CAA GTG CTC AAA CGC-3′; ENBT1 reverse primer, 5′-CTG CCA AGG CTA AGT GCA AGG -3′; GAPDH forward primer, 5′- CGG AGT CAA CGG ATT TGG TCG TAT -3′; GAPDH reverse primer, 5′- AGC CTT CTC CAT GGT GGT GAA GAC -3′.

### Immunofluorescence staining

A specimen of human normal adult liver frozen tissue sections (BioChain Institute, Inc., Newark, CA) was washed three times with PBS, and then incubated for 5 min at room temperature with 3% hydrogen peroxide in methanol to inactivate endogenous peroxidase. After washing three times with PBS, the tissues were incubated for 30 min at room temperature with 5% normal goat serum in PBS to block nonspecific signals. The tissues were washed three times with PBS and then incubated for overnight at 4 °C with the primary antibodies of anti-human SLC43A3 rabbit-polyclonal antibody and anti-human P-glycoprotein mouse-monoclonal antibody (GeneTex Inc.) in Can Get Signal ImmunoStain Solution B (Toyobo). The tissues were washed three times with 0.1% BSA in PBS and those primary antibodies were probed with anti-rabbit IgG F(ab’)2 Alexa Fluor 488 and anti-mouse IgG F(ab’)2 Alexa Fluor 555 (Cell signaling Technology, Beverly, MA), respectively, by incubating for 1 h at room temperature and visualized by using a confocal laser-scanning microscope (LMS510; Zeiss, Jena, Germany).

### Data Analysis

The saturable transport of each substrate, adenine, guanine and hypoxanthine, by ENBT1 was analyzed by assuming Michaelis-Menten type carrier-mediated transport represented by the following equation: *v* = V_max_ × *s*/(K_m_ × *s*). The maximum transport rate (V_max_) and the Michaelis constant (K_m_) were estimated by fitting this equation to the experimental profile of the uptake rate (*v*) versus the substrate concentration (*s*), using a non-linear least-squares regression analysis program, WinNonlin (Pharsight Corp., Mountain View, CA), and the reciprocal of variance as the weight.

When *s* is much smaller than K_m_ (*s*  K_m_), the *v* in the presence of a competitive inhibitor can be described as follows: *v* = *v*_0_/(1 + (*i*/IC_50_)^*n*^). The half-inhibition concentration (IC_50_) was estimated together with the Hill coefficient (*n*) and *v* in the absence of inhibitors (*v*_0_) by fitting this equation to the experimental profile of *v* versus the inhibitor concentration (*i*).

Experimental data are presented as the means ± S.E., and statistical analysis was performed by using Student’s *t* test or, when multiple comparisons were needed, analysis of variance followed by Dunnett’s test, with *p* < 0.05 considered significant.

Western blot analysis, cDNA cloning, and other detailed methods are provided in Supplementary Information.

## Additional Information

**How to cite this article**: Furukawa, J. *et al.* Functional identification of SLC43A3 as an equilibrative nucleobase transporter involved in purine salvage in mammals. *Sci. Rep.* **5**, 15057; doi: 10.1038/srep15057 (2015).

## Supplementary Material

Supplementary Information
