# Corrigendum: Functional identification of SLC43A3 as an equilibrative nucleobase transporter involved in purine salvage in mammals

**DOI:** 10.1038/srep22175

**Published:** 2016-03-04

**Authors:** Junji Furukawa, Katsuhisa Inoue, Junya Maeda, Tomoya Yasujima, Kinya Ohta, Yoshikatsu Kanai, Tappei Takada, Hirotaka Matsuo, Hiroaki Yuasa

Scientific Reports 5: Article number: 15057; 10.1038/srep15057 published online: 10122015; updated: 03042016

In this Article, we failed to indicate that the term ENBT1 was previously used as the name of a putative transporter involved in the dipyridamole-insensitive, purine-selective nucleobase transport system in primary human cardiac microvascular endothelial cells (ref. 11). More recently, the term has also been used to indicate the cellular function of a dipyridamole-insensitive, sodium-independent equilibrative nucleobase transport mechanism for hypoxanthine in particular cells (Bone DB *et al.* Am J Physiol Heart Circ Physiol. 299, H847-856, 2010).

We named SLC43A3 ENBT1 based on its molecular and functional characteristics, and according to the nomenclature of ENT1 (equilibrative nucleoside transporter 1), an alias of SLC29A1. The term ENBT1 used in this article specifically indicates an alias of SLC43A3.

