# Supplementary material for: Functional identification of SLC43A3 as an equilibrative nucleobase transporter involved in purine salvage in mammals
Source: Sci Rep. 2015 Oct 12;5:15057. doi: 10.1038/srep15057 (PMC4796657; doi:10.1038/srep15057)
Supplement: Supplementary Information [file srep15057-s1.pdf]

Functional identification of SLC43A3 as an equilibrative nucleobase transporter involved in purine salvage in mammals

Junji Furukawa, Katsuhisa Inoue, Junya Maeda, Tomoya Yasujima, Kinya Ohta, Yoshikatsu Kanai, Tappei Takada, Hirotaka Matsuo, and Hiroaki Yuasa

Supplementary Methods

Preparation of plasmids

The cDNA of human ENBT1 was cloned from the human lung total RNA (Clontech, Mountain View, CA) by RT-PCR. In brief, an RT reaction was performed using 3 μg of the total RNA, an oligo(dT) primer and ReverTra Ace (Toyobo, Osaka, Japan). The cDNA of ENBT1 was isolated from the obtained cDNA mixture by PCR using KOD FX DNA polymerase (Toyobo) and the following primers: forward primer, 5'- ATT TTC CAA GTG CTC AAA CGC -3'; reverse primer, 5'- CTG CCA AGG CTA AGT GCA AGG -3'. These primers were designed based on the sequence in GenBank (accession no. NM_017611). PCR was performed using the following conditions: 94°C for 2 min; 33 cycles of (i) 94°C for 20 s, (ii) 56°C for 20 s and (iii) 72°C for 1.5 min. The second PCR was performed using the PCR product as a template and a forward primer containing an EcoRI restriction site (underlined), 5'- AGG AAT TCT GCT CAT GGC GG GCC A -3', and a reverse primer containing an XbaI restriction site (underlined), 5'- GCT CTA GAA CTA TGC AAT TGC AGA -3'. Then the amplified product was introduced at the EcoRI and XbaI sites into a mammalian expression vector, pCI-neo (Promega, Madison, WI). The sequence of the final product was determined with an automated sequencer (ABI PRISM 3100; Applied Biosystems, Foster City, CA).

To generate ENBT1 fused with green fluorescent protein (GFP-ENBT1), the cDNA fragments of ENBT1 were prepared by digestion of the pCI-neo-based plasmids carrying ENBT1 cDNA with EcoRI and XbaI, and then introduced into pEGFP-C1 vector (Clontech).

The cDNAs of the APRT and HPRT1 of human were similarly cloned from the human liver total RNA (Clontech) by RT-PCR, using PCR primers designed on the basis of the sequences in GenBank (accession no. NM_000485 and NM_000194, respectively). For the cloning of APRT, the conditions of PCR were as follows: 94°C for 2 min; 33 cycles of (i) 98°C for 20 s and (ii) 68°C for 20 s. The primers for the first PCR were as follows: forward primer, 5'- CTG CCG CTG GCT CTT CGC ACG -3'; reverse primer, 5'- GCA GCC GGT GCC CCT GGT CACT -3'. The second PCR was performed using a forward primer containing an EcoRI restriction site (underlined), 5'- ATG AAT TCA GCC ATG GCC GAC TCC -3', and a reverse primer containing an XbaI restriction site (underlined), 5'-

GAC TCT AGA GAG GCC CTG TGG TCA -3'. For the cloning of HPRT1, the conditions of PCR were as follows: 94°C for 2 min; 33 cycles of (i) 98°C for 20 s, (ii) 57°C for 30 s and (iii) 68°C for 1 min. The primers for the first PCR were as follows: forward primer, 5'- CCT CCT CCT GAG CAG TCA GC -3'; reverse primer, 5'- TTT AGG AAT GCA GCA ACT GAC A -3'. The second PCR was performed using a forward primer containing an EcoRI restriction site (underlined), 5'- AGT GAA TTC CGT TAT GGC GAC CCG CA -3', and a reverse primer containing an XbaI restriction site (underlined), 5'- GCC TCT AGA ACA TTG ATA ATT TTA C -3'.

Western blot analysis

HeLa cells treated for silencing ENBT1 or APRT were washed twice with ice-cold PBS, scraped off, and pelleted by centrifugation at 800 × *g* for 3 min at 4°C. The cell pellet was homogenized by sonication in an ice cold buffer (pH 7.4) containing 250 mM sucrose, 150 mM NaCl, 20 mM Tris-HCl and supplemented with protease inhibitor cocktail (Sigma-Aldrich), and then centrifuged at 2,000 × *g* for 10 min at 4°C. The supernatant was recentrifuged at 15,000 × *g* for 30 min at 4°C, and the resultant pellet was used as the crude membrane fraction sample. The sample (30 μg) was separated on the 10% SDS-polyacrylamide gel by electrophoresis and transferred to Immun-Blot polyvinylidene difluoride membrane (Bio-Rad Laboratories, Hercules, CA). The membrane was blocked with 5% skim milk in Tris-buffered saline (pH 7.4) containing 0.1% Tween 20 (TBS-T) and then probed with primary antibody/anti-human SLC43A3 (ENBT1) rabbit-polyclonal antibody (Atlas Antibodies AB, AlbaNova University Center, Stockholm, Sweden) and anti-human APRT purified rabbit polyclonal IgG (GeneTex Inc., Irvine, CA) at a dilution of 1:200 and 1:1,000, respectively, for overnight at 4°C. After washing three times with TBS-T, the membrane was incubated with secondary antibody/peroxidase-conjugated AffiniPure goat anti-rabbit IgG (H+L) (Jackson ImmunoResearch Laboratories, Inc., West Grove, PA) at a dilution of 1:10,000 for 1 h at room temperature. Then, the expression levels of ENBT1 and APRT were determined by enhanced chemiluminescence using Immobion Western (Millipore, Billerica, MA), according to the manufacturer's instructions.

Table S1. Effect of amino acids on the ENBT1-specific uptake of [^{3}H]adenine

**Amino acid**	**Uptake rate (% of control)**
**Alanine**	**103.0 ± 4.0**
**Histidine**	**101.2 ± 1.8**
**Isoleucine**	**100.4 ± 1.3**
**Leucine**	**94.0 ± 2.5**
**Glutamine**	**104.7 ± 1.3**
**Valine**	**101.1 ± 1.6**
**Methionine**	**95.8 ± 1.4**
**Phenylalanine**	**98.7 ± 1.1**
**Lysine**	**96.6 ± 1.2**
**Asparagine**	**102.7 ± 2.7**
**Arginine**	**95.8 ± 1.7**
**Proline**	**103.4 ± 1.0**
**Tyrosine**	**105.7 ± 1.0**
**Tryptophan**	**95.6 ± 1.8**
**Aspartate**	**99.9 ± 1.3**
**Cysteine**	**104.0 ± 0.8**
**Serine**	**104.6 ± 2.2**
**Glutamate**	**102.7 ± 1.6**
**Threonine**	**99.7 ± 0.7**
**Glysine**	**103.5 ± 1.5**

The ENBT1-specific uptake of [^{3}H]adenine (5 nM) was evaluated at 22°C and pH 7.4 for 40 s in the presence of an amino acid (5 mM) or in its absence. The uptake rate for control was 0.671 ± 0.004 pmol/min/mg protein. Data are presented as the means ± S.E. (n = 4).

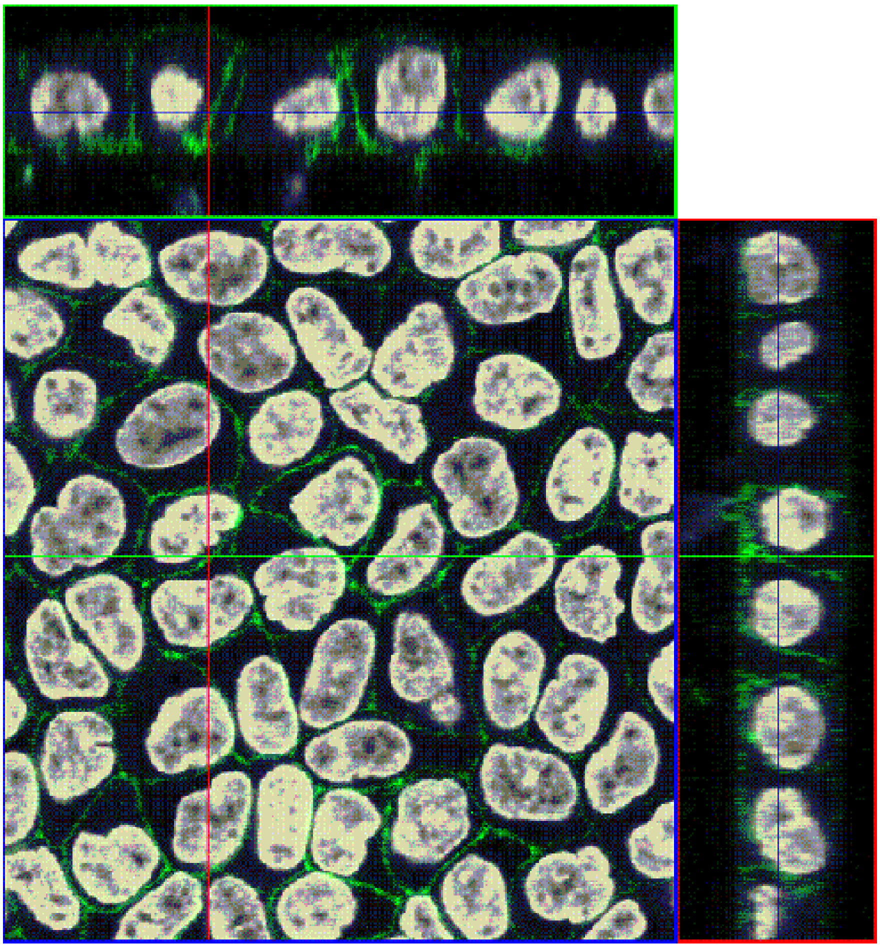

Fig. S1. Localization of GFP-ENBT1 to the basolateral membrane of polarized MDCKII cells. The fluorescent image shows GFP-ENBT1 (green) and nucleus (white).

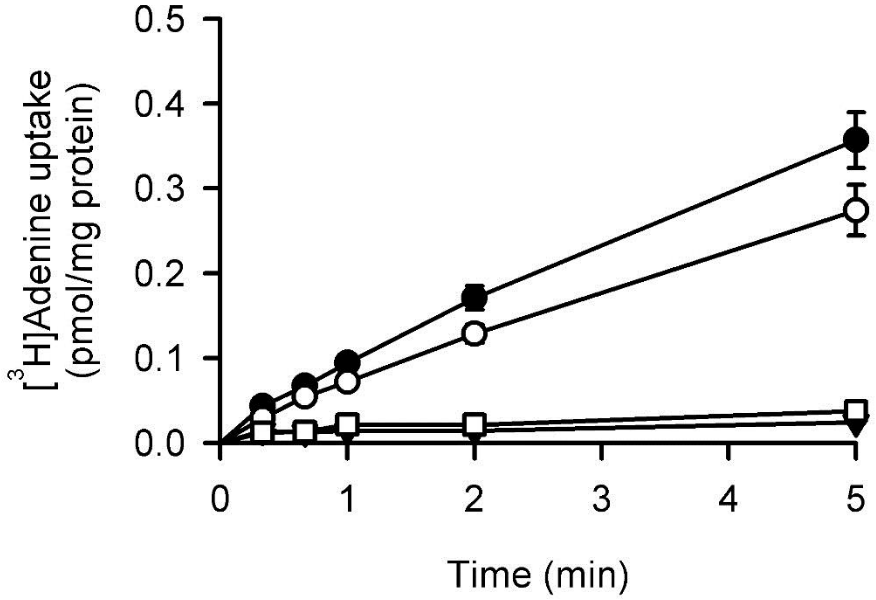

Fig. S2. Time courses of adenine uptake in APRT/HPRT1-deficient A9 cells transiently expressing ENBT1 alone (▼), APRT alone (○) and ENBT1 with APRT(●), and in mock cells (□). The uptake of [^{3}H]adenine (5 nM) was evaluated at 37°C. All the data are presented as the means ± S.E. (*n* = 4).
